# Trends in single-fraction palliative radiotherapy across the COVID-19 pandemic in Japan: a nationwide claims study

**DOI:** 10.1093/jrr/rraf001

**Published:** 2025-01-15

**Authors:** Yutaro Koide, Takahiro Aoyama, Masamune Noguchi, Yurika Shindo, Tomoki Kitagawa, Hidetoshi Shimizu, Shingo Hashimoto, Hiroyuki Tachibana, Takeshi Kodaira

**Affiliations:** Department of Radiation Oncology, Aichi Cancer Center Hospital, Chikusa-ku, Nagoya 464-8681, Japan; Department of Radiation Oncology, Aichi Cancer Center Hospital, Chikusa-ku, Nagoya 464-8681, Japan; Department of Radiation Oncology, Aichi Cancer Center Hospital, Chikusa-ku, Nagoya 464-8681, Japan; Department of Radiation Oncology, Aichi Cancer Center Hospital, Chikusa-ku, Nagoya 464-8681, Japan; Department of Radiation Oncology, Aichi Cancer Center Hospital, Chikusa-ku, Nagoya 464-8681, Japan; Department of Radiation Oncology, Aichi Cancer Center Hospital, Chikusa-ku, Nagoya 464-8681, Japan; Department of Radiation Oncology, Aichi Cancer Center Hospital, Chikusa-ku, Nagoya 464-8681, Japan; Department of Radiation Oncology, Aichi Cancer Center Hospital, Chikusa-ku, Nagoya 464-8681, Japan; Department of Radiation Oncology, Aichi Cancer Center Hospital, Chikusa-ku, Nagoya 464-8681, Japan

**Keywords:** palliative radiotherapy, bone metastases, single-fraction radiotherapy, COVID-19, national claims database

## Abstract

This study aimed to evaluate the recent trends in single-fraction conventional radiotherapy (CRT) as palliative treatment in Japan, using data from the National Database published by the Ministry of Health, Labor, and Welfare. Data from fiscal year (FY) 2014 to FY2022, specifically related to the utilization of single-fraction CRT, were analyzed. Multi-fraction CRT, stereotactic body radiotherapy (SBRT), intensity-modulated radiotherapy (IMRT), and brachytherapy were excluded. The primary outcome was the cumulative and annual number of single-fraction CRT courses. Additionally, quarterly course data from FY2019 to FY2022, the period for which monthly data were available, were assessed to evaluate the impact of the coronavirus disease 2019 (COVID-19) pandemic on single-fraction CRT utilization. Of the total 2 315 607 radiotherapy courses, we identified 33 221 single-fraction CRT courses after excluding multi-fraction CRT (*n* = 1 835 650), SBRT (*n* = 33 935), IMRT (*n* = 332 827), and brachytherapy (*n* = 113 195). The annual number of single-fraction CRT courses increased from 1730 in FY2014 to 5642 in FY2022, with an average annual growth rate of 0.28 (range: −0.07 to 0.65). Outpatient courses significantly increased, particularly from FY2019 onward, surpassing inpatient courses in FY2022 (2914 vs 2728). The highest annual increase was observed in FY2020, particularly from April to December, although this upward trend did not persist in 2021. In conclusion, single-fraction CRT has exhibited a consistent upward trend, highlighting its expanding role in palliative radiotherapy. Although the COVID-19 pandemic temporarily accelerated this trend, its impact has already subsided, with growth rates returning to pre-pandemic levels.

## INTRODUCTION

Conventional fractionated radiotherapy remains the standard approach for various treatment intents. However, in the modern era of radiotherapy, the use of single-fraction conventional radiotherapy (CRT) is largely limited to stereotactic radiotherapy or palliative care. Multiple systematic reviews and meta-analyses have confirmed the efficacy of single-fraction, non-stereotactic, non-intensity-modulated CRT for the palliative treatment of bone metastases [1–6]. To date, no evidence supports the efficacy of single-fraction CRT for definitive treatment purposes. In Japan, the universal health insurance system introduced additional reimbursement incentives for non-stereotactic single-fraction CRT in 2012 compared with multi-fraction CRT. These incentives increased the daily irradiation fee by 20% between fiscal year (FY, April–March) 2012 and FY2013 and by 40% between FY2014 and FY2022 [7–16]. Although this incentive is expected to encourage the use of single-fraction radiotherapy in clinical scenarios that do not require stereotactic body radiotherapy (SBRT) or intensity-modulated radiotherapy (IMRT), the magnitude of this effect needs to be evaluated.

Since the emergence of the coronavirus disease 2019 (COVID-19) pandemic in 2020 in Japan, the Japan Society for Radiation Oncology (JASTRO) recommended the use of hypofractionated radiotherapy for certain cancer sites and a single 8 Gy dose for palliative treatment to reduce patient visits, following similar guidelines from other countries [17–19]. A recent report by Fujita *et al.* demonstrated a brief increase in the use of hypofractionated radiotherapy for breast cancer increased briefly in the early stages of the COVID-19 pandemic. However, unlike other countries, the increase was not sustained in Japan [20]. Although a similar trend is expected in palliative radiotherapy, no studies have assessed the impact of the COVID-19 pandemic on increasing the adoption of single-fraction CRT in a real-world setting.

This study used a national claims database to evaluate the impact of insurance coverages, reimbursement incentives, the COVID-19 pandemic, and recent guidelines or recommendations on the increased usage of single-fraction palliative radiotherapy in Japan.

## MATERIALS AND METHODS

### Study design and endpoint data source

The retrospective study used the National Insurance Claims Database (NDB). This study was approved by our institutional ethics review committee. The JASTRO structural survey results were used to estimate the annual number of patients with bone metastases treated with radiotherapy, as these data could not be retrieved from the NDB database.

The primary outcome was defined as the cumulative and annual number of single-fraction CRT courses between FY2014 and FY2022, based on the NDB database. The secondary outcome was the proportion of single-fraction CRT courses in the estimated palliative radiotherapy for bone metastases based on the JASTRO structural survey results [20–22]. This proportion represents the upper boundary of single-fraction radiotherapy use for the treatment of bone metastases, assuming that all single-fraction radiotherapy in the NDB database was for bone metastases.

### National database analysis

As of August 2024, the NDB database covers 99.9% of Japan’s electronic receipt data from FY2014 to FY2022 and is freely accessible [7–15]. Monthly data became available in the FY2019 database. The NDB database contains data on insured medical practices, including the number of claims, classification codes, practice codes, and insurance scores. However, the database does not provide information on the treatment intent (e.g. curative or palliative) or the target site. The following procedure was used to estimate the number of single-fraction CRT courses.

In this study, ‘radiotherapy with a linear accelerator (other than stereotactic radiosurgery)’ (M001–3, 180 035 310, 8000) was defined as the single-fraction CRT. After excluding treatments covered by the M001–3 code (i.e. stereotactic radiosurgery and radiotherapy [180035310]) in the Japanese universal health insurance system, any external-beam radiotherapy (multi-fraction CRT, conformal-arc therapy, and IMRT) was calculated by multiplying the insurance score by the number of fractions. This indicates that a greater number of fractions results in a higher total income for hospitals. By contrast, the M001–3 code has a fixed insurance score that covers a series of radiotherapy actions (e.g. simulation CT and irradiation technical fees). This fixed score applies regardless of the number of fractions, limiting reimbursement to a single fraction, even if multiple fractions are administered. However, the ‘M001–3, 180035310’ code includes reimbursement incentives for hospitals, which was +20%–40% higher reimbursement than the highest daily insurance score of any multi-fraction CRT using the multi-field technique. Even if the M001–3 code is applied to multi-fraction cases with the lowest daily insurance score (i.e. the one-field technique), common palliative radiotherapy regimens involving the delivery of five or more fractions result in less income for the hospital compared with the multiplied fraction score system. Although non-stereotactic multi-fraction CRT requiring less than five fractions (i.e. two or three fractions) is not entirely excluded from this code, the number of such radiotherapy courses is considered negligible.

During the COVID-19 pandemic, monthly data were used to evaluate detailed quarterly changes in the number of treatment courses. To examine the impact of the pandemic on these changes, we identified the quarterly periods where the rate of increase was at least twice or more than the assumed linear growth rate during the period with available monthly data (FY2019 to FY2022).

### JASTRO structural survey

The JASTRO structural survey was initiated in 1990. It has been conducted every 2 years since then [23]. Unlike the NDB database, the JASTRO structural survey collects responses from radiotherapy facilities, with accuracy dependent on the quality of each facility’s submission. However, this database provides information on treatment intent and target sites and is likely the largest official source of data on the number of bone metastasis irradiations in Japan. The latest available version is from FY2019, based on a survey of 843 facilities conducted in September 2020 [22]. Of the estimated 842 facilities providing radiotherapy in 2019, 734 (87.2%) participated in the analysis. Participation in this survey was mandatory for hospitals seeking accreditation from JASTRO. The survey examined the status of radiotherapy facilities based on regional and prefectural epidemiology, including the number of facilities, types of radiotherapy equipment, facility size, number of treatment plans according to their complexity and annual patient load, number of staff, annual cancer patients according to the patient load of radiation oncology institutions, types of radiotherapies other than external-beam irradiation (e.g. SBRT, IMRT, and particle therapy), target diseases, and treatment sites [20–23].

During the study period (FY2014–FY2022), the JASTRO structural survey published FY2015, FY2017, and FY2019 versions, which documented 29 503, 30 800, and 33 200 palliative radiotherapy courses for bone metastases, respectively. Table 1 presents the estimated number for each FY year, assuming a linear change.

**Table 1:** Recorded and estimated number of palliative radiotherapies for bone metastases

**Year (FY)**	**Recorded number** ^**a**^	**Estimated number (assumed linear change)**
FY2014		28 361
FY2015	29 503	29 457
FY2016		30 553
FY2017	30 800	31 649
FY2018		32 745
FY2019	33 200	33 841
FY2020		34 937
FY2021		36 033
FY2022		37 129

^a^The recorded numbers of FY2015, FY2017, and FY2019 were referred to Japanese Structure Surveys of Radiation Oncology [20–22].

## RESULTS

### Annual numbers of radiotherapy courses

**Fig. 1:**
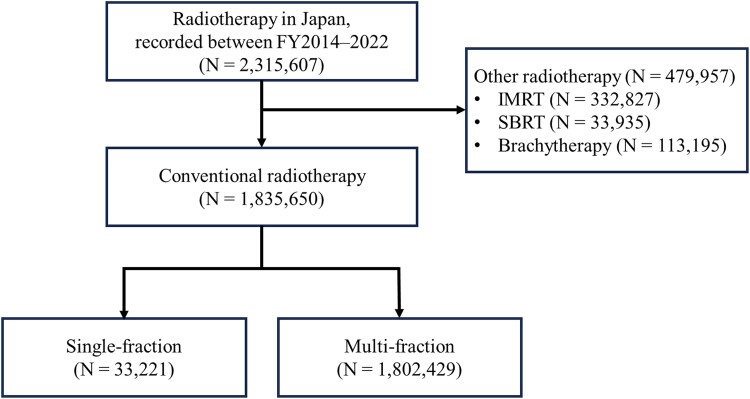
Flow diagram detailing the process of selecting the study cohort.

Figure 1 illustrates the flow diagram of this study. A total of 2 315 607 radiotherapy courses from April 2014 to March 2023 were registered in NDB. After excluding 479 957 other radiotherapy courses (IMRT: *n* = 332 827, SBRT: *n* = 33 935, and brachytherapy: *n* = 113 195), only 1 835 650 CRT courses were analyzed and divided into 33 221 courses of single-fraction CRT and 1 802 429 courses of multi-fraction CRT. The detailed numbers of each radiotherapy type in each year are shown in Table 2.

**Table 2:** Annual number of radiotherapy courses, FY2014–2022

**Year (FY)**	**Overall** ^**a**^	**IMRT + SBRT**	**CRT**	**Single-fraction CRT**		
				**Total**	**Inpatient**	**Outpatient**
2014	245 135	20 471	215 348	1730	1027	703
2015	250 008	25 098	214 822	2115	1238	877
2016	253 408	29 880	212 929	2616	1627	989
2017	251 943	34 434	206 888	3127	1806	1321
2018	258 621	39 161	206 596	3480	2074	1406
2019	268 375	45 654	208 623	4124	2305	1819
2020	260 732	51 656	194 325	5255	2770	2485
2021	262 329	56 546	190 845	5132	2605	2527
2022	265 056	63 862	185 274	5642	2728	2914

IMRT: intensity-modulated radiotherapy; SBRT: stereotactic body radiotherapy; CRT: non-stereotactic, non-intensity-modulated conventional radiotherapy

^a^Overall radiotherapy includes IMRT, SBRT, brachytherapy, and other CRT.

**Fig. 2:**
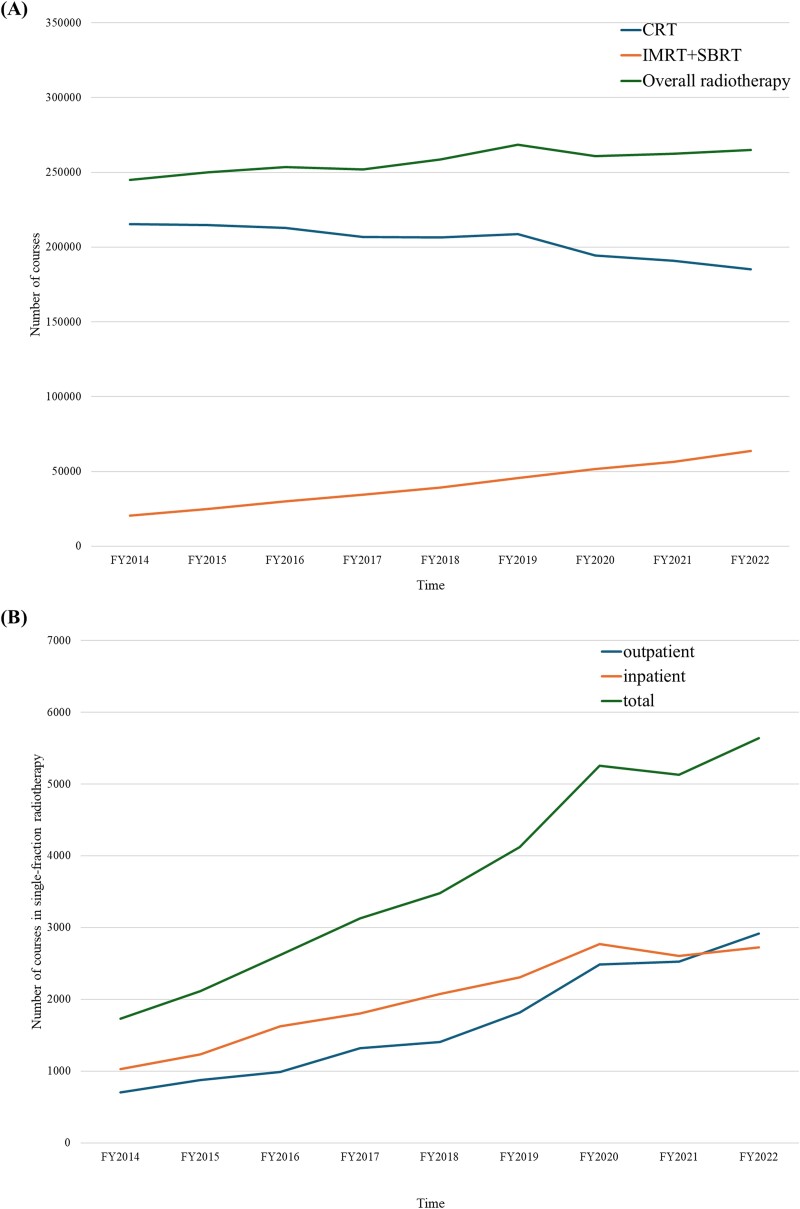
Annual changes in each modality of radiotherapy, FY2014–2022. (A) Shows annual changes in CRT, IMRT, SBRT, and overall radiotherapy. (B) Shows annual changes in single-fraction CRT for inpatients, outpatients, and totals. Mets: metastases, RT: radiotherapy.

Figure 2A displays the annual changes in the total number of radiotherapy courses, IMRT+SBRT, and CRT. The total number of radiotherapy courses remained relatively stable each year, with ⁓250 000 courses reported each year. From FY2014, the number of IMRT+SBRT courses gradually increased from 18 876 (7.8%) to 56 359 (22.4%), whereas the number of CRT courses steadily decreased from 215 348 (89.1%) to 185 274 (73.5%). Figure 2B presents the annual changes in the number of inpatient, outpatient, and overall single-fraction CRT courses. The number of single-fraction CRT courses gradually increased from 1730 to 5642, with an annual average growth rate of 0.28 (range: −0.07 to 0.65). FY2019 showed the highest increase (+0.65), while the following year was the only period that experienced a decrease in the past 9 years (−0.07). When comparing inpatient and outpatient courses, the number of outpatient courses has rapidly increased in recent years, particularly after FY2019, overcoming inpatient courses in FY2022 (2914 vs 2728). Figure 2B also shows the changes in the estimated proportion of single-fraction CRT courses out of the palliative radiotherapy regimens used for the treatment of bone metastases. Compared with the estimated annual number of radiotherapy courses for bone metastasis treatment (Table 1), the upper boundary of the single-fraction CRT rate increased from ≤6.1% in FY2014 to ≤15.2% in FY2022 (Table S1).

### Quarterly numbers of single-fraction conventional radiotherapy courses

**Fig. 3:**
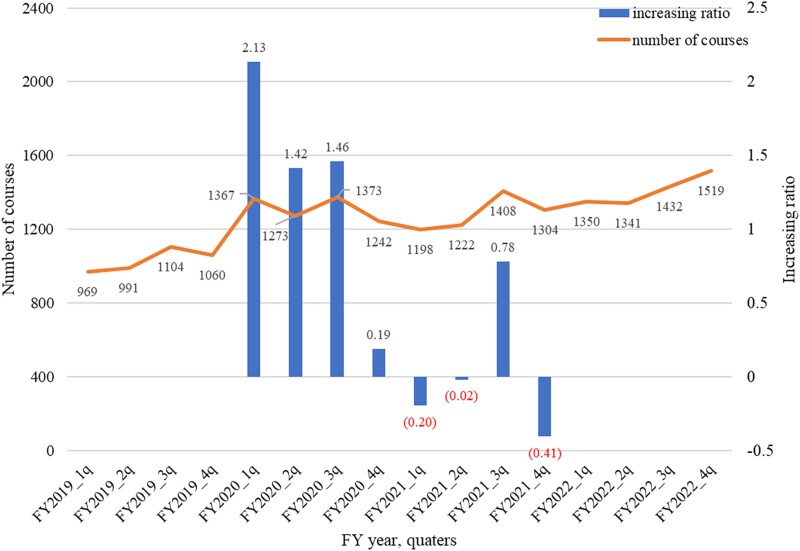
Quarterly changes in single-fraction CRT across the COVID-19 pandemic in Japan. The line shows the quarterly number of courses of single-fraction CRT. The bar graph represents the increasing ratio calculated comparing the quarterly number of CRT courses of the previous year's corresponding quarter. 1q: April to June, 2q: July to September, 3q: October to December, 4q: January to March.

Figure 3 shows the quarterly changes in single-fraction CRT courses between FY2019 and FY2022. An increasing trend was observed in each year compared with the same quarters, with the highest growth rate occurring in FY2020. During the first three-quarters of FY2020 (April to December), the increase was twice or more than the assumed linear growth rate. However, this significant rate of increase did not persist in 2021. The detailed monthly numbers of single-fraction CRT are provided in Table 3.

**Table 3:** Monthly number of single-fraction CRT courses, FY2019–2022

**Month**	**Number of courses, total (outpatient, inpatient)**			
	**FY2019**	**FY2020**	**FY2021**	**FY2022**
April to March	4124 (1819, 2305)	5255 (2485, 2770)	5132 (2527, 2605)	5642 (2914, 2728)
April	391 (165, 226)	430 (212, 218)	420 (201, 219)	471 (229, 242)
May	282 (112, 170)	441 (234, 207)	367 (157, 210)	411 (221, 190)
June	296 (125, 171)	496 (206, 290)	411 (195, 216)	468 (238, 230)
July	345 (162, 183)	479 (231, 248)	419 (217, 202)	426 (202, 224)
August	330 (142, 188)	402 (190, 212)	426 (218, 208)	466 (254, 212)
September	316 (143, 173)	392 (185, 207)	377 (192, 185)	449 (246, 203)
October	312 (150, 162)	445 (189, 256)	447 (228, 219)	472 (245, 227)
November	328 (131, 197)	375 (187, 188)	440 (211, 229)	421 (245, 176)
December	464 (201, 263)	553 (265, 288)	521 (253, 268)	539 (272, 267)
January	310 (126, 184)	371 (174, 197)	405 (200, 205)	430 (228, 208)
February	349 (160, 189)	389 (184, 205)	416 (210, 206)	506 (253, 253)
March	401 (202, 199)	482 (228, 254)	483 (245, 238)	583 (281, 302)

## DISCUSSION

This study is the first to use NDB data to investigate the domestic trend of non-stereotactic, non-IMRT single-fraction radiotherapy in Japan. Although our data showed a continuous increase in the usage of IMRT and SBRT and a decline in the usage of CRT (Figure 2A), the utilization rate of single-fraction CRT is increasing. This suggests that CRT has become increasingly selective and focused on shorter treatment schedules. The NDB is a vast public insurance database that covers over 99.9% of electronic medical receipts managed by the Ministry of Health, Labor and Welfare. As the data are recorded based on insurance practices, it does not indicate whether the treatment of interest is for curative or palliative purposes. However, based on current evidence, single-fraction, non-stereotactic, non-IMRT radiotherapy has demonstrated efficacy primarily in the field of palliative treatment, particularly for painful bone metastases. Single-fraction CRT for non-bone lesions may be selected in real-world practice but rarely reported [6, 24–28]. The proportion of single-fraction CRT was calculated under the assumption, with potential overestimation, that all single-fraction radiotherapies documented in the NDB were administered as treatment for bone metastases. The rate of single-fraction CRT increased from ≤6.1% in FY2014 to ≤15.2% in FY2022.

The socio-epidemiological factor that strongly influenced the shift to single-fraction CRT was the COVID-19 pandemic in 2020 [16]. The monthly NDB data demonstrated a significant increase in the number of single-fraction CRT courses during FY2020, with the number of courses exceeding the average of previous years, particularly in the first 9 months. This increase was likely driven by efforts to minimize patient visits and reduce infection risks during the pandemic. However, this effect did not persist in 2021, as the number of courses temporarily declined, which is consistent with the findings from a study using hypofractionated radiotherapy for breast cancer treatment in Japan [20]. This temporary reduction may reflect the return of some institutions to their usual preference for multi-fraction CRT as the pandemic’s impact lessened in late FY2020. Notably, the increased use of single-fraction CRT during the pandemic coincided with a pre-existing upward trend observed prior to the pandemic, suggesting that factors beyond the pandemic also contributed to this shift. The subsequent increase in the number of single-fraction CRT courses from late FY2021 likely reflects the continuation of this long-term trend.

One possible cause may be related to the domestic insurance system in Japan. Since 2012, the country’s universal health insurance system has provided additional reimbursement incentives for non-stereotactic single-fraction radiotherapy, designating it as a special interest medical practice compared with multi-fraction radiotherapy. The medical practice eligible for this incentive is paid a fixed reimbursement amount for a series of irradiations, regardless of the number or fractions, thereby ensuring the maximum daily reimbursement fee for a single fraction. The reimbursement rates increased by 20% from FY2012 to FY2013 and by 40% from FY2014 to FY2022. The total duration of hospital visits and medical costs for both patients and the insurer may be reduced. Hospitals benefit from the increased daily reimbursement fee for single-fraction radiotherapy compared with multi-fraction radiotherapy, enabling them to allocate additional resources for other treatments (e.g. IMRT or SBRT). This time frame aligns with the NDB data available since 2014, suggesting that the insurance system incentive may be driving a continuous increase in the use of single-fraction radiotherapy. Furthermore, even after the peak of the global COVID-19 epidemic, recent guidelines recommended palliative radiotherapy with a single dose of radiation as the standard of care, rather than multiple doses, which may have further contributed to the continued increase in the use of single-fraction CRT [1–3].

Although this study used the NDB database, several limitations must be acknowledged. First, to calculate the proportion of single-fraction CRT courses out of the estimated annual number of radiotherapies for bone metastasis treatment, we calculated the percentage of single-fraction use in radiotherapy for bone metastases under the assumption that all single-fraction radiotherapy in the NDB was for bone metastases. This assumption likely overestimates the actual proportion as some single-fraction CRT courses may involve treatment of non-bone metastases. However, this percentage represents the upper limit of the expected range, demonstrating that the adoption rate of single-fraction radiotherapy for bone metastases has gradually increased but remains ˂15% in real-world practice. Second, the total number of palliative radiotherapies for bone metastases was estimated by approximating the NDB-measured value based on the results of the JASTRO structural survey. This means that the reliability of data is dependent on the accuracy of the structural survey. Third, this insurance claims study determined the number of radiotherapy courses based on the insurance records, without assessing clinical efficacy or safety. Fourth, as previously discussed, the use of single-fraction radiotherapy showed a continuous increase since FY 2014 due to multiple potential factors. However, the study was unable to assess the impact of each potential factor.

In conclusion, single-fraction CRT has demonstrated a consistent upward trend, highlighting its increasing role in palliative radiotherapy. The COVID-19 pandemic temporarily accelerated this trend, but its impact has subsided, with the adoption rate returning to pre-pandemic levels.

## Supplementary Material

Supplementary_Material_file_1214_rraf001

## Data Availability

Research data are stored in an institutional repository and anonymized numerical data will be shared upon request to the corresponding author.

## AUTHOR RESPONSIBLE FOR STATISTICAL ANALYSIS

Yutaro Koide, MD, PhD., Department of Radiation Oncology, Aichi Cancer Center, Kanokoden 1–1, Chikusa-ku, Nagoya, Aichi, Japan. Email: ykoide@aichi-cc.jp. Tel: (052) 762–6111.
