# Supplementary material for: Trends in single-fraction palliative radiotherapy across the COVID-19 pandemic in Japan: a nationwide claims study
Source: J Radiat Res. 2025 Jan 15;66(1):89–96. doi: 10.1093/jrr/rraf001 (PMC11753830; doi:10.1093/jrr/rraf001)
Supplement: Supplementary_Material_file_1214_rraf001 [file supplementary_material_file_1214_rraf001.docx]

**Supplementary table**

**Table S1. Percentage range of single-fraction radiotherapy in bone metastases radiotherapy**

| **Year (FY)** | **Percentage (upper limit)** |
| --- | --- |
| FY2014 | 6.1% |
| FY2015 | 7.2% |
| FY2016 | 8.6% |
| FY2017 | 9.9% |
| FY2018 | 10.6% |
| FY2019 | 12.2% |
| FY2020 | 15.0% |
| FY2021 | 14.2% |
| FY2022 | 15.2% |

^*^ The percentage of single-fraction radiotherapy in bone metastases radiotherapy was calculated assuming that all single-fraction radiotherapy in the NDB database was for bone metastases, representing the upper limit of the percentage range.
